# Depression or Dementia? The Development of Frontotemporal Dementia in the Shadow of Pseudodementia

**DOI:** 10.1192/j.eurpsy.2025.1722

**Published:** 2025-08-26

**Authors:** M. C. Altınay, F. Ekici

**Affiliations:** 1 Psychiatry, Selcuk University, Konya, Türkiye

## Abstract

**Introduction:**

Pseudodementia presents with symptoms resembling dementia but with cognitive dysfunction improving after depression treatment. Dementia is a progressive, irreversible disorder causing impairment in multiple cognitive domains and daily activities. In the elderly, depressive disorders can manifest as pseudo-forgetfulness due to deterioration of attention functions, while cognitive decline and behavioral changes in dementia may mimic depression. Distinguishing between elderly depression and dementia is challenging because depression often co-occurs with cognitive impairments, and dementia frequently presents with depressive symptoms.

**Objectives:**

This case report aims to highlight the importance of differential diagnosis in cognitive impairments among the elderly by presenting a case where frontotemporal dementia insidiously developed on a background of pseudodementia.

**Methods:**

A 62-year-old woman with a recent history of treatment for depressive symptoms exhibited a gradual decline in planning abilities and behavioral changes over time. Following a non-suicidal jump attempt, she was admitted to the psychiatric ward for diagnostic clarification and treatment. A thorough evaluation of her socio-demographic data, family history, and medical and psychiatric history was conducted.

**Results:**

The patient’s initial complaints began after a psychosocial stressor, including headaches, depressed mood, loss of interest, sleep disturbances, attention difficulties, and increasing forgetfulness. Struggling with daily tasks led her to consult neurology, where age-appropriate atrophy was observed. She was prescribed migraine medication and metformin. Referred to psychiatry, she received sertraline with a preliminary diagnosis of depressive disorder but missed follow-up due to limited social support. Over the next five months, she developed reduced planning abilities, behavioral changes, aimless wandering, inappropriate mood shifts, weakness, and significant functional decline. After a non-suicidal jump attempt, she was admitted to psychiatry. Observations revealed persecutory thoughts and agitation. MRI showed pronounced frontal lobe atrophy inconsistent with her age. Diagnosed with frontotemporal dementia, she was discharged on olanzapine 5 mg and donepezil 10 mg.

**Conclusions:**

This case demonstrates how confounding factors in dementia can adversely affect clinical progression, emphasizing the importance of social support and regular follow-up for accurate diagnosis and treatment. In patients with irregular follow-ups and low social support, conditions like dementia can have dramatic and irreversible courses. Therefore, performing differential diagnosis for dementia in elderly patients with depressive symptoms and reassessing dementia etiologies during follow-ups are vital.

**Disclosure of Interest:**

None Declared

